# Efficacy of the Aim2Be Intervention in Changing Lifestyle Behaviors Among Adolescents With Overweight and Obesity: Randomized Controlled Trial

**DOI:** 10.2196/38545

**Published:** 2023-04-25

**Authors:** Claire N Tugault-Lafleur, Olivia De-Jongh González, Janice Macdonald, Jennifer Bradbury, Tom Warshawski, Geoff D C Ball, Katherine Morrison, Josephine Ho, Jill Hamilton, Annick Buchholz, Louise Mâsse

**Affiliations:** 1 School of Nutrition Sciences, Faculty of Health Sciences The University of Ottawa Ottawa, ON Canada; 2 School of Population and Public Health BC Children's Hospital Research Institute University of British Columbia Vancouver, BC Canada; 3 Childhood Obesity Foundation Vancouver, BC Canada; 4 Department of Pediatrics Faculty of Medicine & Dentistry University of Alberta Edmonton, AB Canada; 5 Department of Pediatrics, Center for Metabolism, Obesity and Diabetes Research McMaster University Hamilton, ON Canada; 6 Cumming School of Medicine, Department of Pediatrics University of Calgary Calgary, AB Canada; 7 Department of Paediatrics, Hospital for Sick Children Toronto, ON Canada; 8 Children's Hospital of Eastern Ontario (CHEO) Ottawa, ON Canada

**Keywords:** mobile health, mHealth, childhood obesity, lifestyle management, adolescents, randomized controlled trial, RCT, mobile phone

## Abstract

**Background:**

Aim2Be is a gamified lifestyle app designed to promote lifestyle behavior changes among Canadian adolescents and their families.

**Objective:**

The primary aim was to test the efficacy of the Aim2Be app with support from a live coach to reduce weight outcomes (BMI *Z* score [zBMI]) and improve lifestyle behaviors among adolescents with overweight and obesity and their parents versus a waitlist control group over 3 months. The secondary aim was to compare health trajectories among waitlist control participants over 6 months (before and after receiving access to the app), assess whether support from a live coach enhanced intervention impact, and evaluate whether the app use influenced changes among intervention participants.

**Methods:**

A 2-arm parallel randomized controlled trial was conducted from November 2018 to June 2020. Adolescents aged 10 to 17 years with overweight or obesity and their parents were randomized into an intervention group (Aim2Be with a live coach for 6 months) or a waitlist control group (Aim2Be with no live coach; accessed after 3 months). Adolescents’ assessments at baseline and at 3 and 6 months included measured height and weight, 24-hour dietary recalls, and daily step counts measured with a Fitbit. Data on self-reported physical activity, screen time, fruit and vegetable intake, and sugary beverage intake of adolescents and parents were also collected.

**Results:**

A total of 214 parent-child participants were randomized. In our primary analyses, there were no significant differences in zBMI or any of the health behaviors between the intervention and control groups at 3 months. In our secondary analyses, among waitlist control participants, zBMI (*P*=.02), discretionary calories (*P*=.03), and physical activity outside of school (*P*=.001) declined, whereas daily screen time increased (*P*<.001) after receiving access to the app compared with before receiving app access. Adolescents randomized to Aim2Be with live coaching reported more time being active outside of school compared with adolescents who used Aim2Be with no coaching over 3 months (*P*=.001). App use did not modify any changes in outcomes among adolescents in the intervention group.

**Conclusions:**

The Aim2Be intervention did not improve zBMI and lifestyle behaviors in adolescents with overweight and obesity compared with the waitlist control group over 3 months. Future studies should explore the potential mediators of changes in zBMI and lifestyle behaviors as well as predictors of engagement.

**Trial Registration:**

ClinicalTrials.gov NCT03651284; https://clinicaltrials.gov/ct2/show/study/NCT03651284

**International Registered Report Identifier (IRRID):**

RR2-10.1186/s13063-020-4080-2

## Introduction

### Background

Although prevalence levels have stabilized over the past decade in Canada, 1 out of 5 and 1 out of 7 children have overweight and obesity, respectively [1]. Multiple health consequences have been associated with a greater extent of adiposity in children, including type 2 diabetes, asthma, joint pain, and mental health conditions [2-5]. Given this and evidence suggesting that childhood and adolescent obesity tracks into adulthood [6,7], there is a need for efficacious, accessible, and engaging lifestyle interventions for hard-to-reach populations, such as adolescents [8].

Current standards of care for childhood obesity management involve family-based interventions that target multiple behaviors associated with obesity (eg, physical activity [PA], diet, and sedentary behaviors) [9,10]. Although such programs have led to short-term improvements in body composition and health behaviors [9,10], high attrition is a common problem reported across interventions [11-13]. Mobile health (mHealth) technologies offer a promising approach to enhance access to weight-management interventions and address potential barriers to care such as the lack of availability for in-person meetings, busy family schedules, and reduced access to health services in rural and remote areas [11,13,14]. In the past decade, the use of web-based or electronic and mobile health platforms (eHealth and mHealth) as modes of delivery for lifestyle interventions has grown substantially. Research examining the potential of mHealth technologies for obesity prevention and treatment suggests their high feasibility and acceptability as both stand-alone and adjunctive interventions for pediatric obesity [14-18]. However, the limited evidence and heterogeneity of studies have made it difficult to draw conclusions on the efficacy and effectiveness of mHealth lifestyle behavior modification interventions in the pediatric context [15,16]. There remains a knowledge gap concerning the efficacy of family-based mHealth interventions for childhood obesity.

### Objectives

To fill this knowledge gap, an mHealth lifestyle behavior intervention (the Aim2Be intervention) was developed to promote healthy behaviors related to nutrition, PA, and screen time among Canadian families [19]. In this paper, we report findings related to the efficacy of the Aim2Be intervention in altering health outcomes and lifestyle behaviors in adolescents with overweight or obesity and their parents. We aimed to (1) test the efficacy of the Aim2Be intervention including support from a live coach to reduce weight outcomes (BMI *Z* score [zBMI]) and improve lifestyle behaviors among adolescents with overweight and obesity and their parents versus a waitlist control group over 3 months (primary aim), (2) compare health trajectories among waitlist control participants over 6 months (before and after receiving access to the app), (3) assess whether support from a live coach enhanced the Aim2Be app impact, and (4) evaluate whether app use influenced changes in health outcomes among intervention participants from baseline to 3 months and then from 3 to 6 months. We hypothesized that participants (child-parent dyads) randomized to the intervention group who had access to the Aim2Be app with live coaching would improve their weight and health behaviors compared with participants who were randomized to a waitlist control group.

## Methods

### Study Design

The trial was prospectively registered in August 2018 (ClincialTrials.gov; NCT03651284) [19] and has been reported in accordance with the CONSORT (Consolidated Standards of Reporting Trials) statement [20]. The CONSORT-EHEALTH (Consolidated Standards of Reporting Trials of Electronic and Mobile Health Applications and Online Telehealth) checklist is available in Multimedia Appendix 1. The protocol for this study has been published previously [19]. This study was a 2-arm parallel randomized controlled trial (RCT) that took place between November 2018 and June 2020. The Aim2Be RCT was based on formative research with parents and adolescents and was piloted before this trial [19]. The primary outcome of the Aim2Be RCT was adolescents’ zBMI scores. Secondary outcomes included lifestyle behaviors (PA, diet, and sedentary activities) among adolescents and parents. Participants were assessed at baseline and at 3 and 6 months using web-based surveys administered through REDCap (Research Electronic Data Capture; Vanderbilt University) [21,22], hosted at the British Columbia Children’s Hospital Research Institute. The staff involved in data collection and analysis were not involved in delivering the intervention.

### Ethics Approval

The evaluation protocol for the study was approved by the Children’s and Women’s Research Ethics Board at the University of British Columbia (H16-03090/H17-02032), the Health Research Ethics Board at the University of Alberta (Pro00076869), the Hospital for Sick Children Research Ethics Board (REB1000059362), the Hamilton Integrated Research Ethics Board (Project #4250), and the Children’s Hospital of Eastern Ontario Research Ethics Board (18/01E). A detailed protocol has been published [19], which is summarized below.

### Study Flow for Intervention and Control Arms

**Figure 1:**
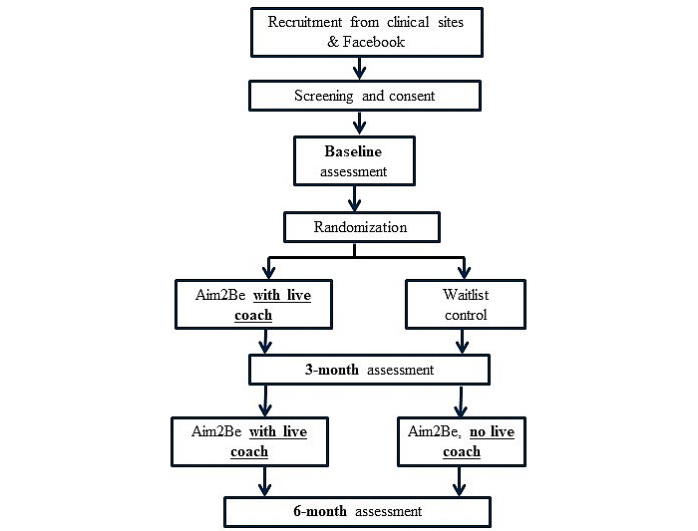
Study flow diagram.

Figure 1 shows an overview of the study flow in this RCT. After screening and baseline assessments, families were randomized into one of the following two groups: (1) an intervention group or (2) a waitlist control group that was given access to the app, but only after 3 months. Families were given access to the Aim2Be app from their home computer or mobile device through an emailed link. The 0- to 3-month period among intervention participants was used to evaluate the efficacy of the intervention in changing zBMI and health behaviors compared with a control group who had no access to the app over 3 months.

Families randomized to the intervention group also received tailored messages from a health coach, with the option of scheduled and unscheduled text support. The health coach was a registered dietitian trained in motivational interviewing and had experience in both pediatric lifestyle management and working with families. This health coach communicated with participants through the in-app text feature, and participants had the option of scheduling a web-based appointment if they wished to do so. The health coach sent an initial contact to all participants enrolled in the intervention group and sent follow-up supportive messages within the app on a regular basis.

Waitlist control participants were put on a waitlist for 3 months, during which they received a brochure with Canadian health recommendations about PA [23], diet [24], screen time, and sleeping habits. Once their 3-month assessment was complete, they were given access to the Aim2Be app for 3 consecutive months but had no access to a live health coach. The 3- to 6-month follow-up period was used to compare the 0- to 3-month period of the intervention condition to evaluate whether additional support from a coach resulted in any additional benefits over time.

### The Aim2Be Intervention

Throughout this manuscript, the Aim2Be intervention included access to the Aim2Be app and the live coach. The theoretical framework guiding the development of the Aim2Be app has been previously published and summarized below [19]. Aim2Be was built on the foundational knowledge learned in the first generation of the program called LiGHT (“Living Green Healthy and Thrifty”) [25]. LiGHT is a 11-module web-based program that integrates lifestyle behavior modification principles with environmental and financial concerns to address childhood obesity among adolescents aged 10 to 17 years and their families [25]. The second generation of the intervention transitioned from an eHealth to an mHealth intervention for the iPhone operating system (iOS) and Android and was renamed Aim2Be.

Aim2Be was developed iteratively to reflect the current recommendations and best clinical practices for pediatric obesity management. Aim2Be became a gamified app that supports youth and their families in initiating sustainable behaviors in 4 primary areas: healthy eating, active living, reducing screen time, and healthy sleeping habits. It retained its focus on linking behaviors with health and living green, as well as adding emphasis on healthy body image and self-esteem. The Aim2Be app uses strategies to strengthen self-regulatory skills through self-guided goals, as well as both planning and self-monitoring of lifestyle behaviors. Strategies used by the various app features were grounded in the behavior change taxonomy by Michie et al [26], which specifies the “active ingredients” of behavior change interventions.

The first version of the app was field-tested for 4.5 months among 301 teens (aged 14-17 years, 33% of whom were overweight or obese) [19]. The quantitative evaluation revealed that teens who were moderately or highly engaged in the app (>30 minutes of total app use) substantially increased their motivation and self-efficacy to improve their dietary habits and sedentary behaviors compared with those with low engagement (≤30 minutes of total app use) [19]. At 4.5 months, teens using the app also substantially increased their previous day’s intake of fruits and vegetables, decreased their consumption of fruit juice, and reduced their screen time [19]. Multiple rounds of qualitative evaluations, including focus groups, 2-week prototype testing, and semistructured interviews, led to numerous improvements in Aim2Be including clarifying the overall purpose of Aim2Be, supplementing the tracking and check-in sections, adding more engaging features, and syncing the app with PA monitoring (ie, Fitbit) [19].

The following 3 versions of the improved version of the app were used in this RCT: a preteen version (for adolescents aged 10-13 years), a teen version (14-17 years), and a companion parent app, with some variations in app features depending on the user type. Once they enrolled in the app, all preteen and teen users were asked to select a personalized animal avatar as part of the onboarding process to personalize their profile. After enrolling in the app, users were offered a selection of aims to address (eg, “Drop sugary drinks” and “Be a healthy family”) and then provided with tasks to help them set incremental goals, plan, and self-monitor their behaviors. Users then progress along their journey by completing quick wins and quizzes. In the preteen and teen versions of the app, users received currency that they could draw on to unlock items (collectibles) and purchase their own adventure stories. All users (preteens, teens, and parents) were provided with tools within the app, including self–check-ins and articles, to further support their journey. In the teen and parent versions of the app, users were able to interact with one another through a moderated social wall, where they could post, comment, and react to each other’s posts. Participants who were randomized to the intervention arm of the trial received an initial in-app text message from the live coach and were sent follow-up supportive messages within the app on a regular basis.

### Participant Recruitment

Participants were recruited from November 2018 to July 2019, and a 6-month follow-up that ended in June 2020. We screened the participants for the trial and later collected the baseline data. Participants were only randomized once they completed all baseline data, so there was a gap in starting the trial from initial recruitment, which explains the longer study period than anticipated in the protocol paper [19]. Families were recruited from the following 6 pediatric weight-management programs across 3 Canadian provinces that typically provide in-person health services for managing childhood obesity: British Columbia Children’s Hospital (Vancouver, British Columbia), Alberta Children’s Hospital (Calgary, Alberta), Stollery Children’s Hospital (Edmonton, Alberta), McMaster Children’s Hospital (Hamilton, Ontario), Hospital for Sick Children (Toronto, Ontario), and Children’s Hospital of Eastern Ontario (Ottawa, Ontario). Clinical sites used a combination of clinic handouts, mail, email, and telephone calls to recruit families. Families waitlisted for in-person weight-management programs and those who declined participation in face-to-face programs were offered participation in the Aim2Be trial as an alternative. Families were provided with an invitational package that described the study and included copies of the consent and assent forms as well as a link to the study website. Families were also recruited using clinic waitlists for in-person weight-management programs and advertisements on Facebook.

### Inclusion and Exclusion Criteria

Interested participants were directed to provide their contact information through an electronic form and were screened by telephone. Families were eligible for inclusion if they had a child between 10 and 17 years old who was overweight or obese (as defined by the age- and sex-specific World Health Organization cutoffs [27]), were capable of reading at the grade 5 level or above, were the primary caregiver for their child, and had a computer or mobile device and internet access at home. Families were ineligible if the child had a diagnosis of type 1 diabetes, anorexia nervosa, or bulimia nervosa; any health condition that restricted the amount or type of PA they could do, the types of food they could eat, or a history of psychiatric problems or substance abuse that could interfere with adherence to the study protocol; if the child was pregnant; or if the child was using other methods of weight-management (eg, participation in another weight-management programs or the use of medication, nutritional supplements, or herbal preparations to lose weight). Only one child per household was eligible to participate in this study.

### Randomization and Blinding

Once eligibility was confirmed, parents and adolescents completed baseline measures, and all participants received a package containing a scale, measuring tape, activity tracker (Fitbit), and brochure with current health recommendations. Once baseline measures were completed, participants were randomized and provided with a CAD $60 (US $46) incentive for e-transfer. A computer-generated randomization schedule was used to allocate participants into blocks of 4, 6, or 8 participants with a randomization ratio of 1:1 [28].

The allocation schedule was concealed in the randomization module of REDCap and only assigned after informed consent and baseline assessments were obtained. Research team members did not enter or modify the allocation schedule; they were exclusively computer generated. Participants were not blinded to their allocation conditions, and allocation assignments were not concealed from researchers at the analysis stage.

### Outcome Measures

#### Primary Outcome: zBMI

Parents were mailed a digital scale (Active Era) and a measuring tape (HDX Corp) with instructions (using the Centers for Disease Control and Prevention home protocol [29]) to accurately measure their child’s height and weight at home. This procedure has been validated to assess children’s height and weight in a home setting [30]. Standardized (zBMI) scores were computed using a Stata macro developed by the World Health Organization, whereby a zBMI >1 and ≤2 SD is classified as overweight and a zBMI >2 SD is classified as obesity [31].

#### Coprimary Outcomes: PA, Diet, and Sedentary Behaviors

PA in adolescents was measured using objective and self-reported data at baseline and at follow-up. The child PA questions were modeled after the International PA and Environment Network questions [32] and inquired about participation in physical education at school in the past week, involvement in team sports, and the number of days of moderate and vigorous PA in the previous week. To assess PA objectively, each participant was mailed their Fitbit Flex 2 (Fitbit Flex 2, Fitbit Inc) [33] at baseline. Wearable devices such as Fitbits have been previously used to objectively measure movement behaviors during PA in lifestyle interventions and have demonstrated reasonable accuracy among adult populations [33]. Children wore the Fitbit for 7-14 days at baseline and at 3 and 6 months, and their daily step count was obtained by our team using Fitabase, a web-based platform designed for research using Fitbits. When processing the Fitbit data for analyses, we chose 1000 steps as an arbitrary cutoff point and considered any days with <1000 steps as invalid and therefore, dropped those days from the analysis. No minimum number of days was required to compute a daily average for each participant (ie, all days with valid Fitbit step counts were used), but 97% of observations included at least 1 weekday (Monday-Friday) and 1 weekend day (Saturday-Sunday). As PA is known to vary between weekends and weekdays, a weighted average number of daily steps was computed for each participant based on whether the reporting day was a weekend or weekday: mean weighted daily steps = 5/7 (mean steps on weekdays) + 2/7 (mean steps on weekends).

Dietary behaviors were measured using a 7-item diet screener (for both adolescents and parents) adapted from the 2016 Canadian Community Health Survey [34], with some questions originating from the Behavioral Risk Factors Surveillance System [35] and the Centers for Disease Control and Prevention National Youth PA and Nutrition Study [36]. Diet screener questions asked about the previous week’s and previous day’s consumption of fruits and vegetables, fruit juices, and sugar-sweetened beverages. To provide a more detailed assessment of dietary intakes, participants were also asked to complete 1 to 3 Waterloo Eating Behavior Questionnaires at baseline, 3 months, and 6 months. This tool is a web-based 24-hour dietary recall developed by the University of Waterloo, which has been validated for use in children and youth [37]. The Waterloo Eating Behavior Questionnaires asks participants to report all foods and beverages (including amounts) consumed during the previous 24 hours from a list of approximately 900 common foods. Data from the 24-hour dietary recalls were then converted into nutrients and food groups servings using the 2007 Canada’s Food Guide food group classification system [38]. Data from these 24-hour dietary recalls were used to estimate mean total daily calories, vegetable and fruit servings, percentage of daily calories from saturated fats, total amount from solid fats (saturated fats and trans fats), total daily intakes of unsaturated fats (in g), total fibers (in g), total sugars (in g), percentage of daily calories from discretionary foods (foods not part of the 4 “core” food groups in the 2007 Canada’s Food Guide), and mean daily calories from sugary beverages (including and excluding 100% fruit juice) at each time point (baseline and 3 and 6 months). To provide an overall measure of adherence to Canadian dietary guidelines [38], an index of overall diet quality (the Canadian Healthy Eating Index [39]) was computed. The C-Healthy Eating Index computes a score from 0 to 100, in which ≤50 is categorized as a poor diet, 50 to 80 as needing improvement, and ≥80 as good [39]. Using cutoffs similar to Barr et al [40], 24-hour recall days were deemed implausible and excluded from the analysis if respondents reported <500 or >6000 kcal/day (6% of the dietary recall days were excluded).

Sedentary behaviors were measured with an adapted version of the assessment of screen time by French et al [41], which has been found to be sensitive to intervention-mediated changes. Two questions asked about the amount of time adolescents spent in front of screens in their free time on weekdays and weekends at each time point. The average weekly screen time variable was computed by taking the weighted average number of minutes spent on screens during weekdays and weekend days combined.

#### Other Measures

Sociodemographic variables were self-reported, and included parental age, sex, race or ethnicity, parental education, marital status, total household income, and recruitment site and method (via social media or a pediatric weight management clinic).

#### Intervention Use

Intervention use and retention or adherence were assessed using web-based data internally collected within the Aim2Be app for parents and adolescents. Intervention use was evaluated using the following indicators: (1) the proportion of participants who downloaded and used the app, (2) total time (in minutes) spent in the app over 3 months, (3) weekly proportion of participants who accessed the app and weekly mean minutes spent on the app over 3 months, and (4) the proportion of participants who used an app feature at least once over 1 month. These analyses included the whole sample, from both the Aim2Be intervention and waitlist control groups (who accessed the app from 3 to 6 months). These measures were computed separately for parents, preteens, and teens because a slightly different app version was developed for preteens [19].

### Power and Sample Size

On the basis of previously published data [42], a sample size of at least 60 families per group was estimated to provide 80% power at an α of .05 to detect a 0.5 decrease in zBMI in the intervention group. We initially targeted a total sample of 200 to account for attrition and missing data and ended up extending to 210 families based on observed attrition. The zBMI was used to calculate the sample size because it is the most difficult variable to change and requires the largest sample size of all primary outcomes. However, to detect a 20% difference in adherence (eg, secondary aims outcome) between the 2 groups (odds ratio of 2.33) at an α of .05, with 80% power using a 1-sided *t* test (1-tailed), 77 families were needed in each group. Additionally, to account for both missing data and attrition, we projected the need for at least 80 families in each group. As the waitlist control group received the intervention after 3 months and further attrition was expected, we required ~100 participants enrolled in each group (accounting for 15% attrition from baseline to 3 months and a small proportion of families who would not download the Aim2Be app). Power calculations were conducted using nQuery software (Statsols).

### Statistical Analyses

All analyses were performed using Stata version 15.1 (StataCorp LLC) and the significance level of all statistical analyses was set at *P*<.05. Intention-to-treat principles were used, with all participants analyzed in the group to which they were randomized, regardless of whether they attended all data collection time points or completed the intervention.

Descriptive statistics were generated to examine the participant characteristics and app use. Student *t* tests (2-tailed; for continuous variables) and chi-square tests (for categorical variables) were used to compare baseline differences between the groups.

The primary analyses for intervention outcomes were performed using linear mixed-effect models to examine changes in child anthropometry, health behaviors, and parental health behaviors, expressed as differences in the means and 95% CI between baseline and 3 months (Aim 1). The *P* value associated with the interaction between group and time was used to determine the statistical significance of any difference between groups over time. Because the between-group differences at baseline were found for 2 outcomes (intake of unsaturated fats and frequency of sugary beverages), we ran models with and without baseline values for these outcomes to determine whether they impacted the results.

Linear mixed-effects models were used to examine changes in health behavior trajectories among participants randomized to the waitlisted control condition before and after being given access to the Aim2Be app (Aim 2). This aim was originally not included in our protocol paper [19]; however, we chose to include this analysis as a secondary exploratory analysis based on previous research that has shown differences in health trajectories among adolescents upon participating in an eHealth intervention [43]. To assess whether additional support from a live coach enhanced the intervention (Aim 3), linear mixed-effect models were used to compare 3-month changes between intervention participants and waitlist control participants who used the app from baseline to 3 months and from 3 to 6 months, respectively. The *P* value associated with the interaction between group and time was used to determine the statistical significance of any difference over time between groups. Finally, to examine whether app engagement (total minutes spent in the app over 3 months) influenced 0 to 3 months and 3 to 6 months changes in health outcomes among intervention participants (aim 4), linear mixed-effect regression models were used. The *P* value associated with the interaction between total app use and time was used to determine the statistical significance of any difference over time between participants who used the app for less than 30 minutes over a 3-month period (“low app users”) and participants who used the app for at least 30 minutes over a 3-month period (“high app users”). The 30-minute cutoff was based on previous dose-response analyses from the formative evaluation phase of the app [19]. All the models included age, sex, race or ethnicity, and educational attainment as covariates. As mixed-effects models perform estimations via maximum likelihood, no imputation was used for these analyses.

Sensitivity analyses were conducted to evaluate differences in outcomes between the control and intervention groups to examine the stability of the results when the intervention sample included only those who used the app for at least 30 minutes in total (from baseline to 3 months). Posttest differences in outcome measures at follow-up between the groups were examined using analysis of covariance. The results are presented for both complete case analyses and multiple imputations. Finally, a portion of the 6-month data (n=32 parent-child dyads) was collected after March 2020 (the beginning of lockdown measures enforced in most Canadian provinces to stop the spread of the COVID-19 virus). Therefore, we conducted exploratory analyses to examine the stability of our results when participants were excluded from our analyses.

## Results

### Characteristics of the Participants

**Figure 2:**
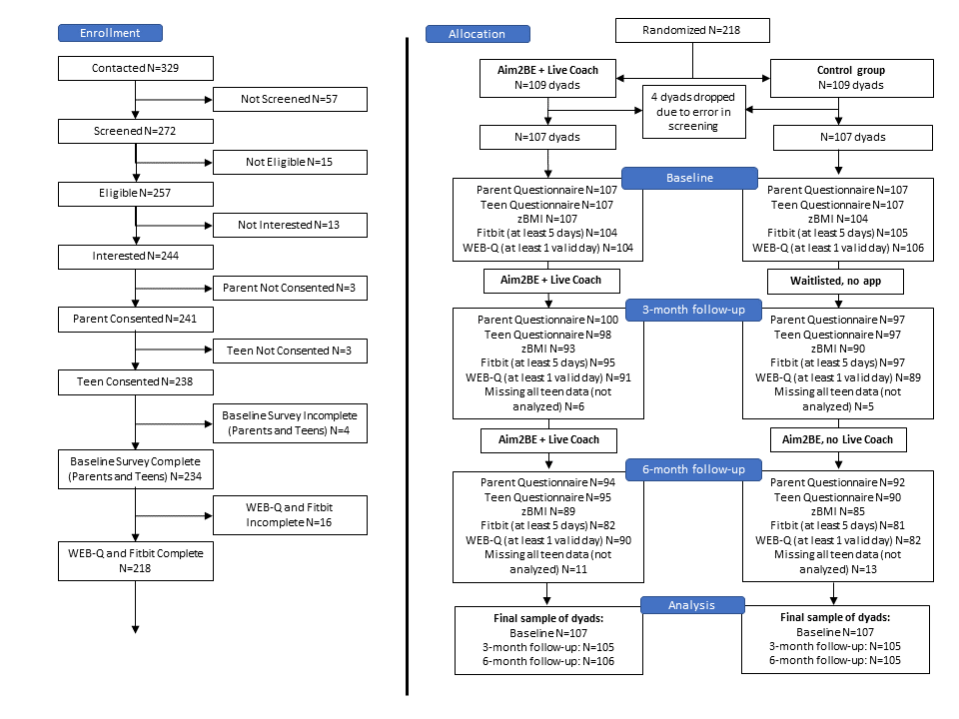
CONSORT (Consolidated Standards of Reporting Trials) flow diagram for participants enrolled in the Aim2Be randomized controlled trial (RCT). WEB-Q: Waterloo Eating Behavior Questionnaires; zBMI: BMI Z score.

Figure 2 shows the study flow of participants. A total of 329 families contacted the research team, and of the 278 families screened for eligibility, 218 agreed to participate, completed all baseline measurements, and were randomized into the study. After randomization, 4 parent-child dyads were dropped from our analyses because within these families, 1 child was assigned to the intervention group and the other sibling was incorrectly assigned to the waitlist control group. Therefore, the final analytic sample comprised 214 parent-child dyads.

The baseline characteristics of participants in the intervention arm are shown in Table 1. In total, 44.4% (95/214) were recruited from clinical sites and 55.6% (119/214) were recruited through social media (ie, Facebook). Randomization was successful in that the participants’ sociodemographic characteristics at baseline did not differ between the intervention and waitlist control groups. Table 2 displays the mean baseline values for health outcomes among the intervention group (n=107 dyads) and waitlist control group (n=107 dyads).

**Table 1:** Sociodemographic characteristics of participants at baseline by intervention arm.

				Intervention (n=107)		Control (n=107)
**Adolescents**						
	Age (years), mean (SD)			12.8 (2.2)		13.1 (2.3)
	Preteens (10-13 years), n (%)			51 (48)		44 (41)
	Teens (14-17 years), n (%)			56 (52)		63 (59)
	Sex (male), n (%)			57 (53.3)		47 (43.9)
**Parents**						
	Age (years), mean (SD)			43.8 (6.8)		44.4 (5.6)
	Sex (female), n (%)			102 (95.3)		96 (89.7)
	BMI^a^ (kg/m^2^), mean (SD)			31.5 (6.6)		32 (8.3)
	Smoking status (% yes), n (%)			14 (13.1)		16 (15)
	**Marital status^b^, n (%)**					
		Married or common-law	79 (74.5)		84 (78.5)	
		Single, separated, or widowed	26 (24.5)		22 (20.6)	
		Prefer not to answer	1 (0.9)		1 (0.9)	
	**Education^b^, n (%)**					
		High school degree or lower	3 (2.8)		11 (10.3)	
		Attended college	54 (50.9)		52 (48.6)	
		Bachelors’ degree or above	49 (46.2)		44 (41.1)	
	**Total household income^b,c^ (CAD $), n (%)**					
		<50,000	19 (17.9)		17 (15.9)	
		50,000 to 99,999	33 (31.1)		33 (30.8)	
		100,000 to 149,999	22 (20.8)		33 (30.8)	
		≥150,000	19 (17.9)		16 (15)	
		Prefer not to answer	13 (12.3)		8 (7.5)	
	**Race or ethnicity^d^, n (%)**					
		White or European	69 (67)		60 (58.3)	
		Aboriginal	5 (4.9)		2 (1.9)	
		East or Southeast Asian	9 (8.7)		3 (2.9)	
		South Asian	3 (2.9)		6 (5.8)	
		Mixed (White and Aboriginal)	6 (5.8)		7 (6.8)	
		Mixed (other combinations)	5 (4.9)		13 (12.6)	
		Other	6 (5.8)		12 (11.7)	
	**Recruitment type** **, n (%)**					
		Clinical sites	48 (44.9)		47 (43.9)	
		Social media (Facebook)	59 (55.1)		60 (56.1)	

^a^Missing data on 11 parents.

^b^Missing data for 1 parent.

^c^The Canadian to US dollar conversion rate at the time of this study was CAD $1 equivalent to US $0.7.

^d^Missing data from 8 parents.

**Table 2:** BMI Z score (zBMI), diet, physical activity, and sedentary behaviors of participants at baseline by intervention arm.

			Intervention, (n=107), mean (SD)		Control, (n=107), mean (SD)
**Adolescents**					
	Standardized zBMI scores	2.89 (0.93)		2.89 (0.95)	
	Total daily energy, kcal	2161 (780)		2006 (688)	
	Healthy Eating Index, total score (range: 0-100 points)	54.6 (12)		53.7 (12.3)	
	Vegetables and fruit, daily servings	3.91 (2.19)		3.50 (2.31)	
	Percent (%) kcal from saturated fat	13.1 (3.4)		12.4 (3.3)	
	Saturated and trans fats, g	32.1 (13.8)		28.9 (12.8)	
	Healthy fat (unsaturated), g	45.3 (18.9)		40 (17.6)	
	Total fiber, g	17.8 (8.7)		16.2 (5.8)	
	Total sugar, g	81 (42.8)		84.4 (47.4)	
	Percent (%) kcal from discretionary foods	20.6 (13.9)		21.3 (13.8)	
	Sugary beverages (includes juice), kcal/day	90 (105)		100 (129)	
	Sugary beverages (excludes juice), kcal/day	58 (84)		76 (116)	
	Frequency of fruit juice, times/day	0.35 (0.54)		0.49 (0.78)	
	Frequency of sugary beverages, times/day	0.41 (0.45)		0.53 (0.51)	
	Physical activity at school, min/week	134 (87)		145 (106)	
	Physical activity outside school, min/week	163 (116)		171 (110)	
	Total physical activity, min/week	298 (146)		317 (161)	
	Fitbit, average daily steps	9209 (3105)		8736 (3508)	
	Screen time, min/day	205 (104)		224 (99)	
**Parents**					
	Frequency of sugary beverages, times/day	0.62 (0.91)		0.40 (0.53)	
	Frequency of fruit juice, times/day	0.14 (0.29)		0.14 (0.24)	
	Fruit and vegetables, daily servings	3.51 (1.53)		3.31 (1.86)	
	Walking, min/day	32 (30)		31 (36)	
	Sitting, min/day	370 (206)		345 (189)	
	Physical activity (moderate and vigorous), min/day	21 (36)		18 (21)	
	Screen time, min/day	147 (89)		147 (75)	

### Primary Analyses

Changes in the outcomes among participants within each trial arm, along with the intervention effects, are shown in Table 3. Changes in adolescent zBMI (primary outcome) were not significantly different between the intervention and control groups (*P*=.51). There were no between-group differences in health behaviors (coprimary outcomes) from baseline to follow-up, except in the control group (mean 3-month change=−42 min/day) in which the screen time was reduced significantly more than the intervention group (mean 3-month change=−2 min/day; *P*=.003). In our sensitivity analyses, no significant differences in any outcome emerged between the control group (n=105 at 3 months) and the reduced sample of intervention participants at 3 months (n=73; Multimedia Appendix 2).

**Table 3:** Effect of the Aim2Be app on health outcomes among adolescents and their parents.

		Mean 3-month changes			Intervention versus control RCT^a^ comparison^b^	
		Intervention (0-3 months)	Control (0-3 months)	β (95% CI)		Group and time interaction*, P* value
**Adolescent outcomes**						
	Standardized zBMI^c^	−0.02	0.03	−0.04 (−0.18 to 0.09)		.51
	Total daily energy, kcal	−182	−101	−91 (−284 to 103)		.36
	Healthy Eating Index, total score (range: 0-100 points)	−1.8	−2.3	0.4 (−3.7 to 4.5)		.86
	Vegetables and fruit, daily servings	−0.38	0.09	−0.31 (−1.11 to 0.49)		.45
	Percent (%) kcal from saturated fat	−1	−0.5	−0.5 (−1.6 to 0.6)		.40
	Saturated and trans fats, g	−4.1	−2.6	−1.7 (−5.8 to 2.4)		.41
	Healthy fat (unsaturated), g	−3.6	−2.5	−1.3 (−7.2 to 4.6)		.67
	Total fiber, g	−0.9	−0.4	−0.3 (−2.6 to 2.1)		.84
	Total sugar, g	−4	−8	2.1 (−10 to 14.1)		.74
	Percent (%) kcal from discretionary foods	−0.4	2.4	−1.8 (−7.2 to 3.6)		.52
	Sugary beverages (includes juice), kcal/day	−17	−17	−8 (−42 to 26)		.64
	Sugary beverages (excludes juice), kcal/day	−1	−16	7 (−28 to 41)		.70
	Frequency of fruit juice, times/day	−0.05	−0.17	0.11 (−0.04 to 0.25)		.15
	Frequency of sugary beverages, times/day	−0.06	−0.06	−0.02 (−0.17 to 0.14)		.84
	Physical activity at school, min/week	−10	−15	5 (−31 to 40)		.80
	Physical activity outside school, min/week	36	25	4 (−31 to 39)		.83
	Total physical activity, min/week	23	21	9 (−42 to 60)		.73
	Fitbit, average daily steps	−638	−532	−137 (−901 to 627)		.73
	Screen time, min/day	−2	−42	41 (14 to 67)		.003
**Parental outcomes**						
	Frequency of sugary beverages, times/day	−0.13	0.05	−0.19 (−0.41 to 0.02)		.07
	Frequency of fruit juice, times/day	0.04	0.03	0.01 (−0.08 to 0.10)		.82
	Fruit and vegetables, daily servings	0.27	0.19	0.09 (−0.35 to 0.53)		.70
	Walking, min/day	8	5	3 (−8 to 14)		.58
	Sitting, min/day	−41	−9	−28 (−79 to 22)		.27
	Physical activity (moderate and vigorous), min/day	5	6	0 (−11 to 10)		.93
	Screen time, min/day	−14	−7	−9 (−32 to 15)		.46

^a^RCT: randomized controlled trial.

^b^Mixed-effects models with maximum likelihood estimation were used to assess the mean estimated difference in the between-group changes in outcomes using 2-way interaction terms (group × time), where group comparisons included intervention and control participants.

^c^zBMI: BMI *Z* score.

### Secondary Analyses

#### Changes Within the Waitlist Control Group (Pre- and Postintervention Changes; Aim 2)

The longitudinal analyses comparing pre- and postintervention changes within the waitlist control participants found a significant decrease in zBMI, meaning that participants experienced a greater decline in zBMI after receiving access to the app as compared with before receiving access to the app (mean difference between phases: −0.10; *P*=.02; Table 4). Compared with before receiving access to the app, waitlist control participants also reported a lower proportion of total daily calories derived from discretionary foods after accessing the app (mean difference between phases: −5.8%; *P*=.03). However, waitlist control participants reported, on average, fewer minutes of PA outside of school (mean difference between phases: −58 min/day; *P*=.001) and more screen time (mean difference between phases: +47 min/day; *P*<.001) after accessing the app than before accessing the app. We examined whether these findings changed after dropping participants who had the 6-month data collected during the COVID-19 pandemic and found no differences, except for the proportion of discretionary calories (*P*=.06) and total weekly PA (*P*=.004).

**Table 4:** Longitudinal changes among control participants before and after receiving access to the app.

		Mean 3-month changes			Trajectory comparison among control participants^a^	
		Control (0-3 months)	Control (3-6 months)	β (95% CI)		*P* value
**Adolescent outcomes**						
	Standardized zBMI^b^	0.03	−0.07	−0.10 (−0.19 to −0.01)		.02
	Total daily energy, kcal	−101	26	126 (−91 to 343)		.25
	Healthy Eating Index, total score (range: 0-100 points)	−2.3	0.8	3.1 (−1.18 to 7.37)		.16
	Vegetables and fruit, daily servings	0.09	0.14	0.06 (−0.69 to 0.80)		.88
	Percent (%) kcal from saturated fat	−0.5	0.3	0.82 (−0.25 to 1.89)		.13
	Saturated and trans fats, g	−2.6	0.35	2.91 (−1.75 to 7.57)		.22
	Healthy fat (unsaturated), g	−2.5	1.5	3.9 (−2.2 to 10)		.21
	Total fiber, g	−0.4	0.11	0.5 (−1.7 to 2.8)		.64
	Total sugar, g	−8	−0.1	7.9 (−5.2 to 20.8)		.24
	Percent (%) kcal from discretionary foods	2.4	−3.5	−5.8 (−11.2 to −0.5)		.03
	Sugary beverages (includes juice), kcal/day	−17	−11	6 (−34 to 45)		.78
	Sugary beverages (excludes juice), kcal/day	−16	−17	−1 (−41 to 39)		.96
	Frequency of fruit juice, times/day	−0.17	−0.02	0.15 (−0.01 to 0.31)		.07
	Frequency of sugary beverages, times/day	−0.06	−0.04	0.02 (−0.18 to 0.22)		.86
	Physical activity at school, min/week	−15	−14	2 (−42 to 45)		.95
	Physical activity outside school, min/week	25	−18	−58 (−92 to −25)		<.001
	Total physical activity, min/week	21	−36	−57 (−115 to 1)		.06
	Fitbit, average daily steps	−532	−903	−371 (−1157 to 415)		.36
	Screen time, min/day	−42	6	47 (21 to 73)		<.001
**Parental outcomes**						
	Frequency of sugary beverages, times/day	0.05	0.02	−0.03 (−0.20 to 0.14)		.75
	Frequency of fruit juice, times/day	0.03	0.10	0.07 (−0.05 to 0.19)		.24
	Fruit and vegetables, daily servings	0.19	0.29	0.10 (−0.34 to 0.55)		.64
	Walking, min/day	5	0	−5 (−16 to 6)		.37
	Sitting, min/day	−9	8	17 (−43 to 77)		.58
	Physical activity (moderate and vigorous), min/day	6	−2	−8 (−17 to 1)		.07
	Screen time, min/day	−7	−7	−5 (−29 to 18)		.67

^a^Longitudinal mixed-effects model with maximum likelihood estimation was used to test for differences in the trajectory of change in outcomes before and after receiving access to the Aim2Be app among control participants.

^b^zBMI: BMI *Z* score.

#### Additional Support From a Live Coach (Aim 3)

No significant between-group differences were found for most outcomes in the analyses that evaluated the effect of additional support from a live coach (Table 5), except for significant time × group interactions for total PA (*P*=.02) and out-of-school PA (*P*=.001). Participants who accessed the app with coaching increased their out-of-school PA (mean difference: +41 min/week from baseline to 3 months), whereas participants who accessed the app without coaching decreased their out-of-school PA (mean difference: −14 min/week from 3 to 6 months). Participants with coaching also increased their total PA (mean difference: +28 min/week from baseline to 3 months), whereas participants who accessed the app without coaching decreased their total PA (mean difference: −36 min/week months; range: 3-6 months). Removing control participants who completed their 6-month follow-up during the pandemic did not change the significance of these findings.

**Table 5:** Effect of the additional support from a live coach on health outcomes among adolescents and parents.

			Mean 3-month changes					Intervention versus waitlisted intervention comparison^a^		
			Intervention (0-3 months)		Control (3-6 months)		β (95% CI)			Group and time interaction, *P* value
**Adolescent outcomes**										
	Standardized zBMI^b^	−0.02		−0.07		0.04 (−0.10 to 0.19)			.55	
	Total daily energy, kcal	−182		26		−196 (−420 to 28)			.09	
	Healthy Eating Index, total score (range: 0-100 points)	−1.8		0.8		−3.1 (−7.4 to 1.2)			.16	
	Vegetables and fruit, daily servings	−0.38		0.14		−0.36(−1.21 to 0.49)			.41	
	Percent (%) kcal from saturated fat	−1		0.3		−1 (−2.2 to 0.1)			.07	
	Saturated and trans fats, g	−4.1		0.35		−4.1 (−8.8 to 0.7)			.09	
	Healthy fat (unsaturated), g	−3.6		1.5		−4.6 (−11.1 to 1.9)			.17	
	Total fiber, g	−0.9		0.11		−0.9 (−3.3 to 1.6)			.49	
	Total sugar, g	−4		−0.1		−3.9 (−16.3 to 8.5)			.54	
	Percent (%) kcal from discretionary foods	−0.4		−3.5		3.8 (−1.9 to 9.5)			.19	
	Sugary beverages (includes juice), kcal/day	−17		−11		−7 (−39 to 26)			.69	
	Sugary beverages (excludes juice), kcal/day	−1		−17		13 (−18 to 44)			.43	
	Frequency of fruit juice, times/day	−0.05		−0.02		−0.03 (−0.18 to 0.11)			.64	
	Frequency of sugary beverages, times/day	−0.06		−0.04		−0.02 (−0.18 to 0.15)			.86	
	Physical activity at school, min/week	−10		−14		−1 (−36 to 35)			.97	
	Physical activity outside school, min/week	36		−18		62 (27 to 97)			.001	
	Total physical activity, min/week	23		−36		62 (9 to 115)			.02	
	Fitbit, average daily steps	−638		−903		254 (−518 to 1027)			.52	
	Screen time, min/day	−2		6		−9 (−36 to 17)			.49	
**Parental outcomes**										
	Frequency of sugary beverages, times/day	−0.13		0.02		−0.16 (−0.36 to 0.04)			.12	
	Frequency of fruit juice, times/day	0.04		0.10		−0.07 (−0.19 to 0.06)			.30	
	Fruit and vegetables, daily servings	0.27		0.29		0.00 (−0.46 to 0.46)			.99	
	Walking, min/day	8		0		9 (−3 to 20)			.14	
	Sitting, min/day	−41		8		−51 (−107 to 5)			.08	
	Physical activity (moderate and vigorous), min/day	5		−2		8 (−3 to 19)			.14	
	Screen time, min/day	−14		−7		−1 (−22 to 19)			.90	

^a^Mixed-effects models with maximum likelihood estimation were used to assess the mean estimated difference in the between-group changes in outcomes using two-way interaction terms (group × time), where group comparisons included the intervention and waitlisted intervention participants.

^b^zBMI: BMI *Z* score.

#### App Use and Changes in Health Behaviors Among Intervention Participants (Aim 4)

App use (minutes spent in the app) did not modify any of the changes in health outcomes for adolescents within the intervention group from baseline to 3 months and then from 3 months to 6 months (Table 6). However, among parents in the intervention group, significant time-by-app use effects were observed for minutes spent walking (*P*=.04) and screen time (*P*=.005). From baseline to 3 months, parents reported a substantial increase in the time spent walking, which then decreased during the 3- to 6-month period. Among parents who reported using the app for at least 30 minutes, the decrease in time spent walking was smaller than that of parents who used the app for less than 30 minutes. From baseline to 3 months, all parents reported reductions in screen time. However, parents who were more engaged with the app reported the smallest reduction in screen time from baseline to 3 months, whereas parents who were less engaged with the app overall reported the largest decline in screen time in this period. Parents across all levels of app use reported similar increases in screen time (on average, by ~10-15 min/day) from 3 to 6 months. These findings remained largely unchanged after dropping participants who provided data after the beginning of the pandemic. However, the time-by-app use interaction for time spent walking by parents was no longer significant (*P*=.06).

**Table 6:** Average 3-month changes and between-phase comparisons in health outcomes among intervention participants who accessed the Aim2Be app for a 6-month period.

			Mean change					Longitudinal mixed analyses^a^		
			0-3 months, (n=105)		3-6 months, (n=106)		β (95% CI)			*P* value^b^
**Adolescent outcomes**										
	Standardized zBMI^c^	−0.02		−0.02		0 (0 to 0)			.29	
	Total daily energy, kcal	−182		−36		0.42 (−0.99 to 1.82)			.56	
	Healthy Eating Index, total score (range: 0-100 points)	−1.8		−0.5		0 (−0.03 to 0.03)			.96	
	Vegetables and fruit, daily servings	−0.38		−0.05		0 (0 to 0.01)			.69	
	Percent (%) kcal from saturated fat	−1		0.12		0 (−0.01 to 0.01)			.96	
	Saturated and trans fats, g	−4.1		−0.2		0 (−0.03 to 0.03)			.91	
	Healthy fat (unsaturated), g	−3.6		−0.2		0.02 (−0.03 to 0.07)			.43	
	Total fiber, g	−0.9		0		0 (−0.01 to 0.02)			.81	
	Total sugar, g	−4		−1.2		0.01 (−0.07 to 0.09)			.81	
	Percent (%) kcal from discretionary foods	−0.4		0		0 (−0.04 to 0.04)			.92	
	Sugary beverages (includes juice), kcal/day	−17		9		−0.09 (−0.29 to 0.11)			.36	
	Sugary beverages (excludes juice), kcal/day	−1		6		−0.13 (−0.31 to 0.06)			.17	
	Frequency of fruit juice, times/day	−0.05		0.07		0 (0 to 0)			.95	
	Frequency of sugary beverages, times/day	−0.06		0		0 (0 to 0)			.16	
	Physical activity at school, min/week	−10		3		0.06 (−0.16 to 0.28)			.57	
	Physical activity outside school, min/week	36		−14		0.12 (−0.13 to 0.37)			.36	
	Total physical activity, min/week	23		−12		0.18 (−0.15 to 0.51)			.28	
	Fitbit, average daily steps	−638		−704		−0.57 (−6.39 to 5.25)			.85	
	Screen time, min/day	−2		−1		−0.09 (−0.26 to 0.09)			.33	
**Parental outcomes**										
	Frequency of sugary beverages, times/day	−0.13		0.01		0 (0 to 0)			.41	
	Frequency of fruit juice, times/day	0.04		−0.05		0 (0 to 0)			.66	
	Fruit and vegetables, daily servings	0.27		−0.11		0 (0 to 0.01)			.89	
	Walking, min/day	8		−9		0.13 (0 to 0.26)			.04	
	Sitting, min/day	−41		7		−0.29 (−0.80 to 0.22)			.27	
	Physical activity (moderate and vigorous), min/day	5		−2		0.12 (−.01 to 0.24)			.06	
	Screen time, min/day	−14		5		−0.30 (−0.52 to −0.09)			.005	

^a^Linear mixed-effects model with maximum likelihood estimation was used to assess mean estimated differences in baseline to 3-month and 3- to 6-month changes, while controlling for app use (total number of minutes in the app), adolescents’ age and sex (for adolescents’ outcomes only), parental age and sex (for parental outcomes only), parental race, and educational attainment.

^b^*P* value from interaction terms (time × minutes in the app) were used to test for differences in changes in outcomes between time periods across various levels of app use among participants.

^c^zBMI: BMI *Z* score.

### Intervention Use

Among the 214 parents randomized to either the intervention or waitlist intervention groups, 190 (88.7%) were enrolled in the app. Most parents accessed the app via an iOS or Android system, but 1021 out of 4567 sessions (22% of all parent sessions) were accessed via a web-based (computer) platform. Among the 190 enrolled parents, 182 (96%) logged into the app for at least one session.

Of the potential 99 preteens, 87 (88%) were enrolled in the app and among the 87 enrolled, 85 (98%) logged for at least 1 session in the app. Out of the potential 115 teens, 102 (89%) enrolled in the app, and among those 102 enrolled, 91 (89%) logged for at least one session in the app. Most adolescents accessed the app via an iOS or Android system, but 582 out of 4877 sessions (12% of all adolescent sessions) were accessed via a web-based platform.

By week 2, the percentage of participants who used the app at least once had decreased to <60% for both preteens and parents and <50% for teens (Multimedia Appendix 3). Retention rates remained >40% for preteens and parents (during the first 5-6 weeks) compared with teens (which lasted only 2 weeks), indicating a sharper decline in teen engagement compared with that of parents and preteens. Multimedia Appendix 4 shows the prevalence of preteens, teens, and parents who engaged at least once with a given app feature over 3 months (intervention and waitlist control groups combined). Relatively few participants engaged with the behavioral features of the app (eg, aims and tasks). Apart from completing self–check-ins, popular app features (features used by >70% of adolescents) consisted of collecting items and interacting with an app-based Chatbot (“Aim2Be-bot”). Among the intervention participants, just less than half of the parents and teens (50/107, 46.7% and 27/56, 48%, respectively) and over half (30/51, 58%) of the preteens responded to a chat message from the live coach.

## Discussion

### Study Overview

Our study is the first RCT to assess the efficacy of an app-based lifestyle intervention on zBMI and lifestyle behaviors among overweight and obese Canadian youth that was mostly smartphone-based with no clinical in-person component. This study found no evidence of an effect on zBMI scores or any coprimary outcomes (PA, diet, and sedentary behaviors) for intervention participants compared with those of a control group over a 3-month period. Our primary analyses revealed no significant differences in zBMI or any of the health behaviors between the intervention and control groups. Secondary exploratory analyses revealed the following mixed findings: among waitlist control participants, zBMI, discretionary calories, and PA outside of school declined, whereas screen time increased after receiving app access compared with before receiving app access. Overall, we did not find evidence of additional benefits in terms of giving participants access to a live coach, and app use did not modify any outcomes among adolescents in the intervention group.

### Relevance of the Findings

In contrast with 2 recent systematic reviews and meta-analyses that pooled findings from eHealth interventions targeting children and adolescents [14,44], we did not find a major effect on BMI or zBMI. However, the pooled effect sizes from the 2 reviews [14,44] were small and the clinical importance of these effects remains unclear. Our null findings align with an earlier review that reported a nonsignificant effect on children’s BMI or zBMI based on a pooled analysis of 5 trials that all targeted parents as agents of change [16]. Several systematic reviews examining the efficacy of eHealth and mHealth interventions in changing lifestyle behaviors have highlighted how engagement can be a major issue in eHealth and mHealth interventions [45,46]. The lack of an intervention effect on our primary outcome (zBMI) and coprimary outcomes (health behaviors) in the RCT could be a result of several factors. First, the sample of parent-adolescent dyads recruited in this trial constitutes among the most difficult and difficult-to-reach population. Recruitment for about half of the participants occurred at pediatric weight-management clinics, and many sites offered Aim2Be app access as an alternative to in-person treatment. Therefore, it is possible that many of the families enrolled had lower motivation or readiness to change compared with families ready to commit to a face-to-face and a more time-intensive program. Although the intervention did not focus on weight but rather on promoting healthy behaviors for families, it is possible that many of the study participants were already experiencing weight stigma and discrimination, which could have impacted their psychological health before the intervention and, therefore, resulted in low levels of engagement. Second, our process evaluation revealed that relatively few participants engaged with the more “active ingredients” (the behavioral features of the app—aims and tasks). Although many features were incorporated into the app to promote engagement, it is unclear whether these features (while enjoyable and fun for preteens and teens) might have distracted them from the features meant to promote and support behavior change. Taken together, these findings speak to the difficulties inherent in mHealth and eHealth behavior change interventions, particularly those related to maintaining participant engagement.

When we corrected for app use in our RCT analyses, our findings related to the efficacy of the app did not change, suggesting that the total number of minutes spent in the app over 3 months did not predict changes in health behaviors over time. However, it is worth noting that a crude measure of engagement (total time spent in the app) might not provide a nuanced and comprehensive picture of what aspects of the intervention “works” or leads to changes in health outcomes. Factor mixture modeling of web-analytics data from a previous version of the app among 301 teens who used the app for 4.5 months revealed distinct engagement profiles ranging from “uninvolved” teens to “dabblers,” “engaged,” and finally “keeners” [47]. “Keeners” had the highest use of all app features and improved on most mediators of behavior change and increased their vegetable and fruit intake. Similarly, separate analyses characterizing app user typologies among adolescents and parents enrolled in the current RCT have shown that using the active ingredients of the app is necessary to obtain major improvements in weight and health behaviors among youths [48].

Our findings suggest that the Aim2Be app might have had some effects on the waitlisted control participants. Although initially challenging to understand, there may be some plausible explanations for these findings. There could be seasonality effects, as the randomization of participants for this RCT could not be spread over the course of a full year. It is also possible that participants randomized to the waitlist intervention arm (control participants), who remained in the study to receive the intervention from 3 to 6 months, had greater motivation or readiness for change compared with participants randomized to the intervention arm. In a separate exploratory analyses, we found that adolescents in the waitlisted control group reported decreased total daily calories and energy from solid fats (saturated fats and trans fats) during the waitlist period (0-3 months; *P*<.05 from paired *t* tests for both outcomes), indicating that these participants had already begun to change some of their dietary behaviors before receiving access to the app. These findings reiterate the importance of considering motivation and readiness for change before engaging in lifestyle interventions.

Our findings suggest that providing participants with access to a web-based live coach did not enhance the intervention. It is worth highlighting that relatively few participants engaged with the live coach via SMS text messaging and even fewer participants set up web-based appointments. Although approximately half of the individuals randomized to the app with live coach conditions sent at least one chat message to the live coach, none of the parents set an internet-based face-to-face appointment, making it difficult to assess whether the support of the live coach yielded additional benefits overall. Although intervention participants exposed to the app with a live coach experienced improvement in self-reported (but not objectively measured) PA compared with waitlisted intervention participants, these differences could be attributed to seasonality effects, as the randomization of participants for this RCT could not be spread over the course of a full year.

Previous research has shown the key role of parents in supporting health behavior changes through the household environment [50,50]. In an eHealth intervention study conducted among teens in Canada, the household environment (specifically parenting practices, parenting styles, and household income) predicted a large proportion of variance in adolescents’ adherence to the intervention [51]. Another analysis using data from the above trial also found that the parental adherence rate was considerably associated with the adolescent participation rate [52]. Despite this RCT having a parent companion app, our findings suggest that the intervention was not successful at actively engaging parents and supporting health behavior changes among parents. Parents may have emphasized supporting their adolescents without making any changes to their own behaviors, as found in a separate profiling study conducted with parent-child dyads participating in this RCT [48]. Separate analyses are underway to explore the potential mediators and predictors of engagement, and whether engagement is related to health outcomes.

### Limitations

This study had several limitations. The first and likely the most relevant being the generalizability of the findings given who were eligible to participate in this study (parent-adolescent dyads in Canada with either overweight or obesity). Therefore, these results may not apply to other populations (children without overweight or obesity) and adolescents outside Canada. Second, we found that preteens and teens predominantly spent time in the gamified components of the app instead of using its active ingredients (such as setting aims and completing tasks). There was an effort to strengthen access to the gamified or “fun” elements of the revised version of the app for this trial, which might explain why there was no association in the evaluation phase as compared with an earlier formative evaluation phase. Third, we could not control for seasonality effects, as the recruitment of participants for this trial could not occur over a year because of limited funds. Fourth, participants in both conditions were provided with their Fitbit at baseline (shortly after randomization), which could have resulted in an early PA intervention effect among the waitlisted control participants. Fifth, this study presented only the findings related to intervention efficacy, and there is a growing consensus on the need to explore metrics beyond efficacy, such as cost-effectiveness, reach, and engagement, which could allow a broader examination of the impact of such interventions [53].

### Conclusions

In summary, the Aim2Be trial was not effective in improving zBMI, PA, diet, or screen time over a 3-month period among overweight and obese Canadian children compared with those of a waitlist control group. However, secondary analyses revealed some beneficial effects of the intervention among waitlisted control participants who experienced a decline in zBMI and discretionary calories after receiving app access compared with those from before receiving app access. Future studies should explore the mediators of changes in lifestyle behaviors and identify strategies to increase app user engagement with the “active ingredients” of interventions.

## Appendix

Multimedia Appendix 1 CONSORT-eHEALTH checklist (V 1.6.1).

Multimedia Appendix 2 Sensitivity analyses exploring 3-month changes in health behaviors between the control and intervention participants who used the app for at least 30 minutes over 3 months.

Multimedia Appendix 3 Preteen, teen, and parent app use by time over 3 months.

Multimedia Appendix 4 Preteen, teen, and parent engagement with each app feature over 3 months.

## Abbreviations

CONSORT: Consolidated Standards of Reporting Trials
CONSORT-EHEALTH: Consolidated Standards of Reporting Trials of Electronic and Mobile Health Applications and Online Telehealth
iOS: iPhone operating system
mHealth: mobile health
PA: physical activity
RCT: randomized controlled trial
REDCap: Research Electronic Data Capture
zBMI: BMI Z score
