# Supplementary material for: Efficacy of the Aim2Be Intervention in Changing Lifestyle Behaviors Among Adolescents With Overweight and Obesity: Randomized Controlled Trial
Source: J Med Internet Res. 2023 Apr 25;25:e38545. doi: 10.2196/38545 (PMC10170359; doi:10.2196/38545)
Supplement: Multimedia Appendix 2 [file jmir_v25i1e38545_app2.docx]

**Multimedia appendix 2**

Table. Sensitivity analyses examining 3-month changes in health behaviours between control and intervention participants who used the app for at least 30 min over 3 months

|  | BASELINE | | | | | | 3 MONTHS | | | | | | CCA | MI |
| --- | --- | --- | --- | --- | --- | --- | --- | --- | --- | --- | --- | --- | --- | --- |
|  | **Control, n=107** | | | **Intervention app users who used the app >30min over 3 months, n=73** | | | **Control, n=105** | | | **Intervention app users who used the app >30min over 3 months, n=73** | | | **Postest differences** | **Postest differences** |
|  | **N** | **Mean** | **SD** | **N** | **Mean** | **SD** | **N** | **Mean** | **SD** | **N** | **Mean** | **SD** | **P value** | **P value** |
| Children |  |  |  |  |  |  |  |  |  |  |  |  |  |  |
| BMI z scores^1^ | 104 | 2.9 | 1 | 73 | 2.8 | 1 | 88 | 2.9 | 0.9 | 68 | 2.8 | 0.9 | 0.262 | 0.238 |
| Total energy, daily (kcal) | 106 | 2006 | 688 | 73 | 2197 | 675 | 88 | 1926 | 629 | 68 | 1947 | 642 | 0.999 | 0.921 |
| Healthy Eating Index (HEI), total score (range: 0-100 points) | 106 | 54 | 12 | 73 | 55 | 13 | 88 | 52 | 13 | 68 | 55 | 14 | 0.698 | 0.736 |
| Fruits & vegetables (daily servings) | 106 | 3.5 | 2.3 | 73 | 4.1 | 2.3 | 88 | 3.5 | 2.4 | 68 | 3.5 | 2.8 | 0.305 | 0.543 |
| Percent (%) calories from saturated fat | 106 | 12.4 | 3.3 | 73 | 13.5 | 3.6 | 88 | 12 | 3.8 | 68 | 12.3 | 3.3 | 0.102 | 0.484 |
| Unhealthy fat (saturated and trans) (g.) | 106 | 28.9 | 12.8 | 73 | 33.2 | 13 | 88 | 26.8 | 13.5 | 68 | 27.9 | 13.5 | 0.407 | 0.728 |
| Healthy fat (unsaturated) (g.) | 106 | 40 | 17.6 | 73 | 46 | 17.1 | 88 | 38.1 | 17.4 | 68 | 40.1 | 20.7 | 0.762 | 0.990 |
| Total fiber (g.) | 106 | 16.2 | 5.8 | 73 | 17.9 | 8 | 88 | 15.6 | 6.5 | 68 | 16.7 | 8.1 | 0.373 | 0.627 |
| Total sugar (g.) | 106 | 84.4 | 47.4 | 73 | 81.2 | 44.6 | 88 | 77.4 | 43.7 | 68 | 72.3 | 34.4 | 0.755 | 0.725 |
| Percent (%) of daily calories from discretionary foods | 106 | 21.3 | 13.8 | 73 | 19.7 | 14.8 | 88 | 22.7 | 15 | 68 | 17.9 | 15.7 | 0.862 | 0.835 |
| Calories (kcal) from all sugary beverages including juice | 106 | 100 | 129 | 73 | 88 | 97 | 88 | 93 | 126 | 68 | 66 | 86 | 0.200 | 0.329 |
| Calories(kcal) from sugar-sweetened beverages, excludes juice | 106 | 76 | 116 | 73 | 53 | 75 | 88 | 66 | 108 | 68 | 54 | 81 | 0.596 | 0.631 |
| Daily frequency of fruit juice (times per day)^1^ | 106 | 0.5 | 0.8 | 72 | 0.3 | 0.6 | 95 | 0.4 | 0.5 | 70 | 0.3 | 0.5 | 0.115 | 0.561 |
| Daily frequency of sugary beverages (times per day) | 107 | 0.5 | 0.5 | 73 | 0.4 | 0.4 | 95 | 0.5 | 0.6 | 70 | 0.3 | 0.3 | 0.957 | 0.943 |
| Fruit and vegetables (daily servings), excludes fruit juice and fried potatoes | 107 | 2.6 | 1.7 | 73 | 3.2 | 1.9 | 94 | 3.1 | 1.8 | 70 | 2.9 | 1.9 | 0.687 | 0.735 |
| Physical activity at school (min/week) ^1^ | 102 | 144.7 | 106.4 | 69 | 139.4 | 87.8 | 87 | 127.4 | 128.4 | 65 | 119.2 | 99 | 0.873 | 0.215 |
| Physical activity outside school (min/week) ^2^ | 107 | 171 | 110.2 | 73 | 172.6 | 118.3 | 95 | 195.2 | 107.5 | 70 | 197.1 | 118.3 | 0.620 | 0.760 |
| Total Physical activity (min/week) | 102 | 317 | 161.2 | 69 | 315.1 | 147 | 87 | 332.2 | 181.7 | 65 | 319.5 | 143.4 | 0.993 | 0.981 |
| Fitbit - average daily steps | 107 | 8735.7 | 3508.3 | 72 | 9345.7 | 3174.7 | 98 | 8327.8 | 3149.4 | 71 | 8641.7 | 3477.9 | 0.406 | 0.715 |
| Screen time (min/day) | 107 | 223.6 | 99.3 | 71 | 189.3 | 107.8 | 95 | 180.4 | 96.6 | 69 | 189.4 | 101.8 | 0.559 | 0.589 |
| Parents |  |  |  |  |  |  |  |  |  |  |  |  |  |  |
| Daily frequency of sugary beverages (times per day) | 107 | 0.4 | 0.5 | 68 | 0.6 | 0.8 | 95 | 0.5 | 0.7 | 66 | 0.6 | 0.8 | 0.949 | 0.840 |
| Daily frequency of fruit juice (times per day)^1^ | 106 | 0.1 | 0.2 | 68 | 0.1 | 0.3 | 95 | 0.2 | 0.4 | 65 | 0.2 | 0.3 | 0.784 | NA |
| Fruit and vegetables (daily servings), excludes fruit juice and fried potatoes | 107 | 3.3 | 1.9 | 68 | 3.7 | 1.5 | 95 | 3.5 | 2 | 66 | 4 | 1.5 | 0.054 | 0.092 |
| Walking: daily average (min) | 106 | 31.1 | 36 | 67 | 31.7 | 27.9 | 94 | 36.1 | 37.9 | 65 | 39.7 | 37 | 0.169 | 0.379 |
| Sitting: daily average (min) | 107 | 344.6 | 188.7 | 68 | 369.3 | 215.2 | 95 | 328.1 | 224.7 | 66 | 321.4 | 225.4 | 0.431 | 0.576 |
| Physical activity, moderate and vigorous^1^ | 107 | 18 | 21.3 | 64 | 21.1 | 30.1 | 91 | 23.9 | 33.6 | 65 | 26.5 | 35.5 | 0.921 | NA |
| Screen time, min/day (weekly average) | 104 | 147.1 | 75.5 | 67 | 136.8 | 82.6 | 93 | 142.9 | 88.5 | 65 | 128 | 87.6 | 0.255 | 0.333 |

CCA, Complete Case Analyses; MI, multiple imputation.

N=107 families in Aim2Be and n=107 families in the Control group. Differences in outcome measures at follow-up between groups were examined using analysis of covariance (ANCOVAs) for most continuous outcomes.

^1^ For these outcomes, some of the basic assumptions for ANCOVAs models were not met (significant baseline*group interactions were identified for these outcomes). Therefore, the p-value for the Postest group difference with CCA represents the p value of the interaction between time and group using linear mixed effect models. When ANCOVA assumptions were not met with imputed data, then no imputation is reported (NA).

**^2^** Model was also adjusted for baseline physical activity at school.
