# Supplementary figures and images for: Efficacy of the Aim2Be Intervention in Changing Lifestyle Behaviors Among Adolescents With Overweight and Obesity: Randomized Controlled Trial
Source: J Med Internet Res. 2023 Apr 25;25:e38545. doi: 10.2196/38545 (PMC10170359; doi:10.2196/38545)

**Multimedia Appendix 3**


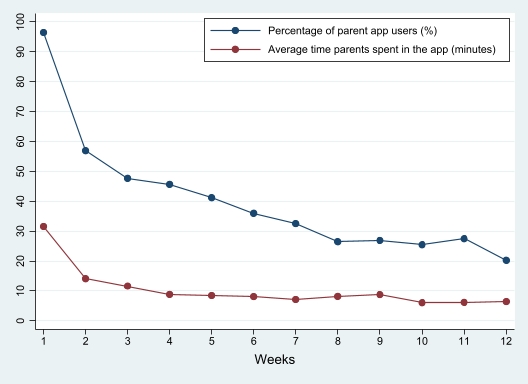

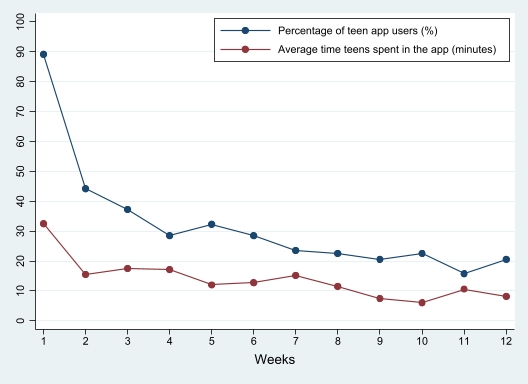

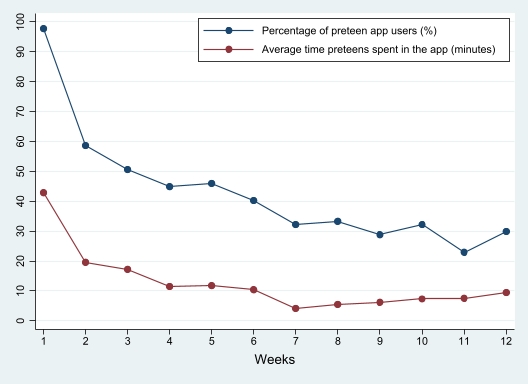
Figure. Preteen, teen and parent app use by time over 3 months

Supplement: Multimedia Appendix 3 [file jmir_v25i1e38545_app3.docx]
