# Supplementary material for: Efficacy of the Aim2Be Intervention in Changing Lifestyle Behaviors Among Adolescents With Overweight and Obesity: Randomized Controlled Trial
Source: J Med Internet Res. 2023 Apr 25;25:e38545. doi: 10.2196/38545 (PMC10170359; doi:10.2196/38545)
Supplement: Multimedia Appendix 4 [file jmir_v25i1e38545_app4.docx]

Multimedia Appendix 4


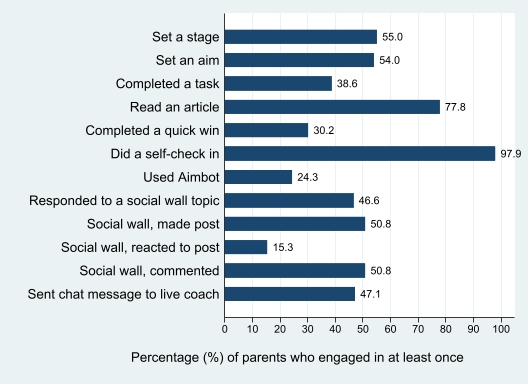

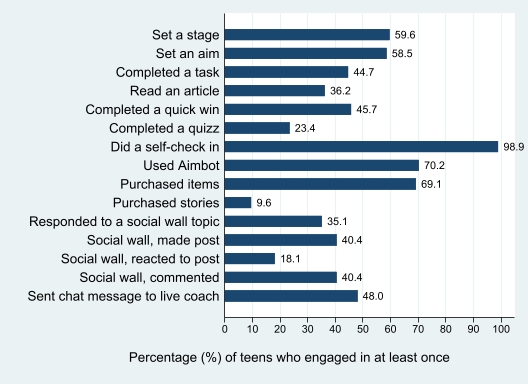

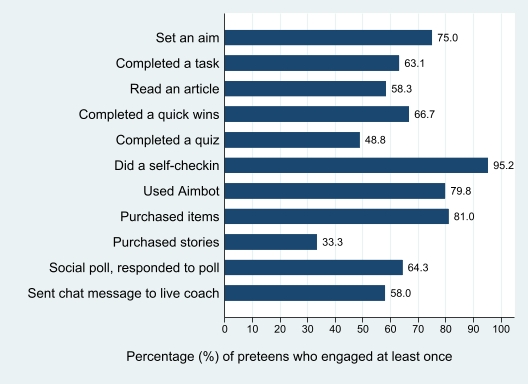
Figure. Preteen, teen and parent engagement with each app feature over 3 months
